# Metformin alleviates bevacizumab-induced vascular endothelial injury in mice through growth differentiation factor 15 upregulation

**DOI:** 10.22038/IJBMS.2023.72759.15827

**Published:** 2024

**Authors:** Liqiang Chen, Yajuan Yin, Chunmiao Liu, Junying Liu, Mingqi Zheng, Yida Tang, Qing Yang, Jing Liu, Fan Chen, Lanbo Liu, Gang Liu

**Affiliations:** 1 Department of Cardiovascular, The Fourth Hospital of Hebei Medical University, Shijiazhuang, China; 2 Department of Cardiovascular, The First Hospital of Hebei Medical University, Shijiazhuang, China; 3 Department of Obstetrics,The Fourth Hospital of Shijiazhuang,Shijiazhuang, China; 4 Department of Pathology, The Fourth Hospital of Hebei Medical University, Shijiazhuang, China; 5 Department of Cardiology, Peking University Third Hospital, 49 Huayuanbei Road, Haidian District, Beijing 100191, China; 6 Department of Cardiology, Tianjin Medical University General Hospital, 154 Anshan Road, Heping District, Tianjin 300052, China; # These authors contributed equally to this work

**Keywords:** Bevacizumab, Growth differentiation-factor 15, Metformin, Mouse, PI3K/AKT/FOXO/PPARγ- Signaling pathway, Vascular injuries

## Abstract

**Objective(s):**

Bevacizumab is a commonly used anticancer drug in clinical practice, but it often leads to adverse reactions such as vascular endothelial damage, hypertension, arterial and venous thrombosis, and bleeding. This study investigated the protective effects of metformin against bevacizumab-induced vascular injury in a mouse model and examined the possible involvement of GDF15/PI3K/AKT/FOXO/PPARγ signaling in the effects.

**Materials and Methods:**

C57 male mice were purchased. To investigate metformin, the mice were assigned to the saline, bevacizumab (15 mg every 3 days), metformin (1200 mg/day), and bevacizumab+metformin groups. To investigate GDF15, the mice were assigned to the siNC+bevacizumab, siNC+bevacizumab+metformin, siGDF15+bevacizumab, and siGDF15+bevacizumab+metformin groups. Histological staining was used to evaluate vascular injury. Flow cytometry was used to evaluate apoptosis. ELISA was used to measure plasma endothelial injury markers and proinflammatory cytokines. qRT-PCR and western blot were used to determine the expression of GDF15 and PI3K/AKT/FOXO/PPARγ in aortic tissues.

**Results:**

Metformin alleviated bevacizumab-induced abdominal aortic injury, endothelial cell apoptosis, and systemic inflammation in mice (all *P<*0.05). Metformin up-regulated GDF15 expression and PI3K/AKT/FOXO/PPARγ signaling in the abdominal aorta of mice treated with bevacizumab (all *P<*0.05). siGDF15 abolished the vascular protective and anti-inflammatory effects of metformin (all *P<*0.05). siGDF15 suppressed PI3K/AKT/FOXO/PPARγ signaling in the abdominal aorta of mice treated with bevacizumab (all *P<*0.05).

**Conclusion:**

Metformin attenuates bevacizumab-induced vascular endothelial injury, apoptosis, and systemic inflammation by activating GDF15/PI3K/AKT/FOXO/PPARγ signaling.

## Introduction

Despite the great achievements of cancer therapy in recent years, cancer remains the first or second cause of death in people aged 30-69 years in 134 countries and is the third or fourth cause of death in other countries (1). Cancer-related vascular disorders, such as cardiovascular diseases and stroke, are important contributors to cancer’s high mortality rate (2-4). Angiogenesis participates in tumor progression, and inhibiting angiogenesis is a recognized strategy against several cancers (5-8). On the other hand, inhibiting angiogenesis can also damage the endothelial function of normal blood vessels (9, 10), and endothelial dysfunction is involved in the development of atherosclerosis and cardiovascular diseases (11, 12). Bevacizumab is a targeted cancer therapy that inhibits the vascular endothelial growth factor (VEGF) and is widely used against cancer (13). Despite its efficacy against cancer, bevacizumab increases the risk of vascular diseases, including arterial thrombosis, hemorrhage, and hypertension (14-16). Bevacizumab was also associated with a higher risk of cardiac and cerebral ischemia (17). In addition, the incidence of bevacizumab-induced arterial thrombosis is 3.8%-5% (18, 19), and the incidence of bevacizumab-induced venous thrombosis can reach 12% (16). Therefore, there is an urgent need to find a solution to reduce bevacizumab-induced vascular injury.

Metformin is an antidiabetic medication that benefits the cardiovascular system (20). Metformin protects the cardiovascular system by improving mitochondrial function, glucose and lipid metabolism, and autophagy of the vascular endothelial cells (21). In addition, available data suggests that metformin has some effects against cancer by inhibiting mTOR and cancer cell proliferation (22). Even though the use of metformin against cancer itself is becoming controversial (23), metformin could be used in cancer patients to protect against treatment-induced endothelial dysfunction and prevent cardiovascular events (24). Indeed, adding metformin to bevacizumab therapy improves the outcomes of tumor-bearing mice and cancer patients (25-27), but the underlying mechanisms remain unknown. 

Growth differentiation factor 15 (GDF15) is a stress response cytokine belonging to the transforming growth factor β superfamily. GDF15 is a protective cytokine that prevents atherosclerosis development and vascular endothelial injury (28, 29). Metformin stimulates GDF15 expression and secretion in diabetic patients (30, 31). Our previous *in vitro* study has shown that metformin alleviates bevacizumab-induced apoptosis, vascular endothelial injury marker expression, and proinflammatory marker expression of human umbilical vein endothelial cells (HUVECs) by up-regulating GDF15 expression and activating PI3K/protein kinase B (AKT)/forkhead box O (FOXO)/peroxisome proliferator-activated receptor γ (PPARγ) signaling (32). Still, the effect of metformin on bevacizumab-induced vascular endothelial injury *in vivo* remains unexplored. 

This study aimed to investigate the protective effects of metformin against bevacizumab-induced vascular injury in a mouse model and examine the possible involvement of GDF15/PI3K/AKT/FOXO/PPARγ signaling in the effects. The results suggest that metformin may prevent bevacizumab-induced vascular injury by activating GDF15/PI3K/AKT/FOXO/PPARγ signaling, thus reducing the risk of vascular diseases in cancer patients treated with bevacizumab.

## Materials and Methods

***Animals***

Twenty-four male C57 mice (6-8 weeks old, 22-24 g) were purchased from Beijing Weitong Lihua Laboratory Animal Technology Co., Ltd (certificate of conformity No. 1100112011028991; license No.: SCXK (Beijing) 2016-0006). The animals were fed under pathogen-free conditions with a 12:12 hr light-dark cycle at 21-25 ^°^C. 

An adeno-associated virus vector AAV-GP-1 expressing small interfering RNA against mouse GDF15 (siGDF15:5’-GCAGGCAACTCTTGAAGACTT-3’; batch #200817DZ) was purchased from GenePharma (Suzhou, China). A vector expressing a scrambled RNA (5’-TTCTCCGAACGTGTCACGT-3’) was used as a negative control (siNC; batch # E10BZ; GenePharma). 

In order to investigate the effect of metformin on bevacizumab-induced vascular injury, the mice were divided into four groups and received the corresponding treatments, including saline, bevacizumab (15 mg via intraperitoneal injection, once every 3 days; Sinopharm, China), metformin (1200 mg via gavage, once daily; Med Chem Express, China), and bevacizumab+metformin. 

In order to investigate the role of GDF15 in the vascular protective effect of metformin, the mice were divided into four groups and administered siNC+bevacizumab, siNC+bevacizumab+metformin, siGDF15+bevacizumab, or siGDF15+bevacizumab+metformin. siRNAs (0.2 ml/mouse) were administered via tail vein injection every 2 weeks. 

Bevacizumab was administered in a clinically used injectable dosage form (100 mg/4 ml) diluted to 40 ml (i.e., 10-fold dilution) for convenient use in mice (15 mg/kg every 3 days). Metformin was dissolved in distilled water, and mice were gavaged (1200 mg once daily).

The doses of bevacizumab and metformin used in this study were the maximum tolerated and effective doses determined by preliminary animal experiments. In the preliminary experiments, mice were divided into three groups treated with bevacizumab for 7 weeks: 5 mg/kg every 3 days, 10 mg/kg every 3 days, and 15 mg/kg every 3 days. The 15-mg/kg group showed the most significant abdominal aortic endothelial damage. Then, the combination of bevacizumab and metformin was investigated in mice treated with bevacizumab 15 mg/kg every 3 days. The mice received metformin 300 mg/kg/day, 600 mg/kg/day, 1200 mg/kg/day, and 2400 mg/kg/day. All mice in the metformin 2400 mg/kg/day died within 3 days. Therefore, metformin 1200 mg/kg/day was used in this study. These preliminary results are supported by the literature (33-36).

All animals were sacrificed by cervical dislocation 7 weeks after treatment. The orbital blood was obtained for ELISA. The abdominal aorta was collected and stored at -80 ^°^C until use. This study was approved by the Ethics Committee of the Fourth Hospital of Hebei Medical University (IACUC-4th hosHebmu). All experiments were carried out following the ARRIVE guidelines.

***Flow cytometry***

Mouse aortic tissue was homogenized and digested with 0.2% trypsin on a shaking platform for 20 min at 37 ^°^C. After three cycles of trypsinization, the cell suspension was filtered through a 200-mesh filter and centrifuged at 1500 rpm for 5 min. The supernatant was removed, and the cells were resuspended in PBS and centrifuged at 1500 rpm for 5 min. After two cycles of PBS washing, cell apoptosis was examined by incubation with Annexin V-FITC (5 μl; Solarbio, Beijing, China) and propidium iodide (5 μl; Solarbio) in the dark at room temperature for 30 min, followed by analysis using a flow cytometer (BD Biosciences, USA).

***Quantitative reverse transcription PCR (qRT-PCR)***

Total RNA was isolated from the abdominal aorta sample using the TRIzol reagent (Ambion, USA), followed by cDNA synthesis using a HiFiScript gDNA Removal cDNA synthesis kit (CoWin BioSciences, Cambridge, MA, USA). PCR was conducted using ChemoHS qPCR Mix on a Bio-Rad PCR instrument (Hercules, CA, USA). β-actin was used as an internal reference. The sequences of gene-specific primers (GenePharma) are summarized in Table 1. The relative expressions of the genes were calculated using the 2^-ΔΔCt^ method, 

***Western blot analysis***

Total proteins were isolated from 100 mg of abdominal aorta tissue by centrifuging the tissue lysates for 15 min at 14,000 ×g and 4 ^°^C. The protein concentration was measured using a protein concentration quantifier (22331 Hamburg type, Eppendorf, Hamburg, Germany). Equal amounts of proteins (30-50 μg) were separated by SDS-PAGE and transferred to PVDF membranes (MilliporeSigma, Burlington, MA, USA). The membranes were blocked with Tris-Tween 20 buffer containing 5% bovine serum albumin for 2 hr and then incubated with primary antibodies (Table 2) overnight at 4 ^°^C. The membrane was washed with PBS-T 3×10 min and incubated with secondary antibodies (Table 2) for 1.5 hr at room temperature. The protein bands were visualized using enhanced chemiluminescence and quantified using the Tanon Gis software. 

***Hematoxylin and eosin (H&E) staining***

Mouse aortic tissue samples were fixed in 4% paraformaldehyde at room temperature for 24 hr, embedded in paraffin, cut into 5-μm thick sections, and stained with H&E. The sections were observed and photographed using a BX43 optical microscope (Olympus, Japan).

***ELISA***

Orbital blood samples were collected from the mice 7 weeks after drug treatment. The plasma concentrations of E-selectin (CD62E; #KE10036), endothelin-1 (ET-1; #E-EL-M2730c-96T), thrombomodulin (TM; #E-EL-M1140c-48T), tumor necrosis factor (TNF)-α (#KE10002), and interleukin (IL)-6 (#KE10007-96T) were measured using ELISA kits from Proteintech (China) following the manufacturer’s instructions. Plasma von Willebrand factor (vWF) level was determined using an ELISA kit from Elabscience Biotechnology (#E-EL-M1247c-48T; China) following the manufacturer’s protocol. The absorbance was measured at 450 nm wavelength using an Elx800 microplate reader (BioTek, USA).

***Statistical analysis***

All experiments were repeated three times with three biological replicates. Data were expressed as the mean±standard deviation and analyzed using SPSS 26.0 (IBM, Armonk, NY, USA). Comparisons among multiple groups were conducted using a one-way analysis of variance and Tukey’s multiple comparisons test. Comparisons between the two groups were performed using the unpaired t-test. A *P*-value<0.05 was considered statistically significant.

## Results

***Metformin alleviates bevacizumab-induced abdominal aortic injury, endothelial cell apoptosis, and systemic inflammation in mice***

**Figure 1:**
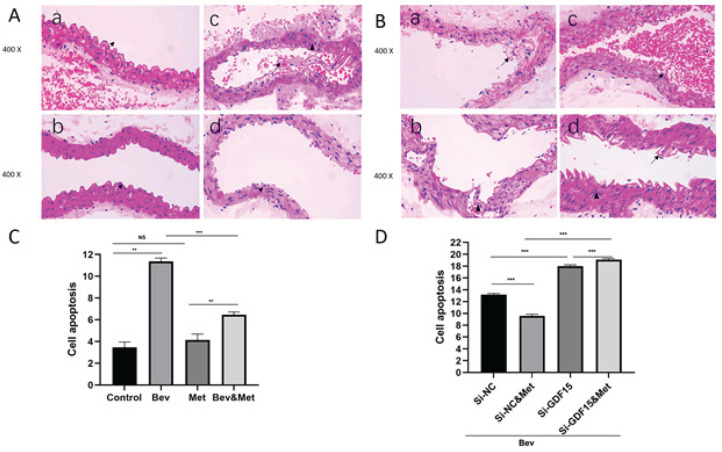
Effect of metformin or GDF15 silencing in bevacizumab-induced vascular endothelial injury and endothelial cell apoptosis in mouse abdominal aorta

**Figure 2:**
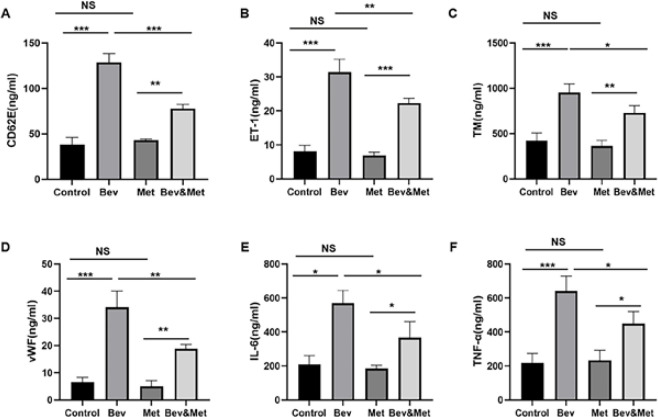
Effect of metformin on bevacizumab-induced changes in plasma levels of vascular endothelial injury markers and proinflammatory cytokines

In order to explore the effect of metformin on bevacizumab-induced vascular injury, we treated male C57 mice with metformin and bevacizumab, alone or in combination, and collected the abdominal aorta and blood samples at 7 weeks after treatment. H&E staining showed that bevacizumab administration resulted in arterial intima dilatation and tortuosity, rupture of the inner elastic fiber layer, and red blood cell infiltration into the subintimal layer of the artery (Figure 1A, a and b). No significant changes were observed in the metformin group compared with the control group (Figure 1A, c). Compared with bevacizumab alone, the addition of metformin significantly alleviated the abnormalities induced by bevacizumab (Figures 1A, b, and d). Flow cytometry showed that bevacizumab significantly promoted apoptosis of vascular endothelial cells, which was effectively reversed by metformin (Figure 1C). ELISA (Figure 2A-F) revealed that bevacizumab dramatically elevated the plasma levels of endothelial injury markers (CD62E, ET-1, TM, and vWF) and proinflammatory cytokines (IL-6 and TNF-α) compared with control (all *P<*0.001). Metformin significantly abrogated the changes induced by bevacizumab (all *P<*0.05). These data suggest that metformin protects the arteries from bevacizumab-induced endothelial injury and inflammation. 

***Metformin up-regulates GDF15 expression and PI3K/AKT/FOXO/PPARγ signaling in the abdominal aorta of mice treated with bevacizumab***

**Figure 3:**
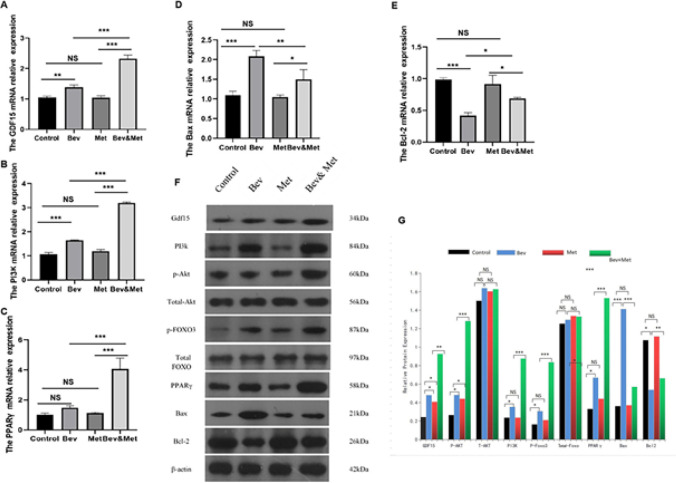
Effect of metformin on bevacizumab-induced alterations in the expression of GDF15 and PI3K/AKT/FOXO/PPARγ in mouse abdominal aorta

We have previously found that metformin protects HUVECs from bevacizumab-induced apoptosis *in vitro* by enhancing GDF15 expression and PI3K/AKT/FOXO/PPARγ signaling (32). In order to investigate the underlying mechanism of metformin’s vascular protective activity *in vivo*, we determined the expression of GDF15, PI3K/AKT/FOXO/PPARγ, and apoptotic markers in the abdominal aorta tissue samples from the mice. qRT-PCR showed that in the presence of bevacizumab, metformin considerably enhanced GDF15, PI3K, and PPARγ mRNA expression (Figure 3A-C). Western blot analysis showed that metformin promoted GDF15, P-AKT, PI3K, P-FOXO3, and PPARγ protein expression in the presence of bevacizumab (all *P<*0.01) (Figure 3F-G). In addition, metformin abolished bevacizumab-induced alterations in Bax and Bcl-2 expression (Figure 3D-G). These results suggest that GDF15 up-regulation and PI3K/AKT/FOXO/PPARγ activation are involved in the protective effect of metformin against bevacizumab-induced vascular injury and endothelial cell apoptosis *in vivo*.

***siGDF15 abolishes the vascular protective and anti-inflammatory effects of metformin***

**Figure 4:**
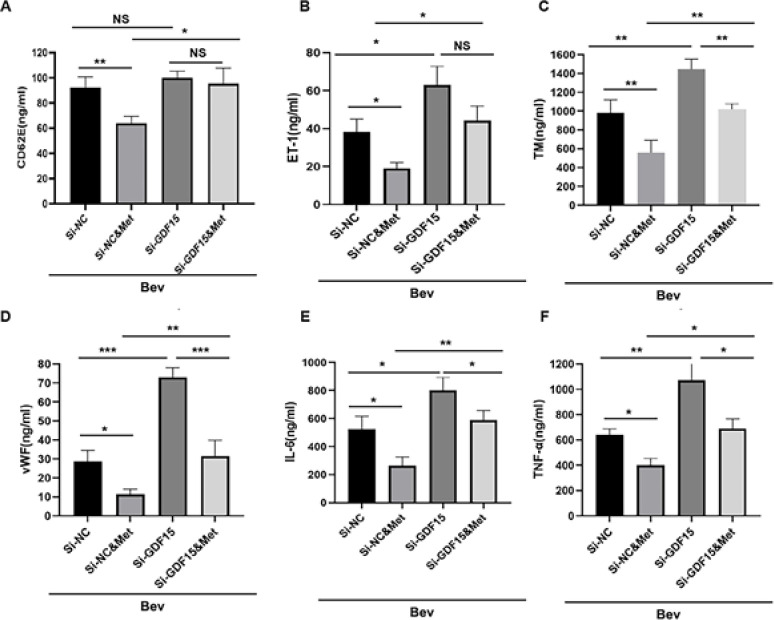
Knockdown of GDF15 reversed the effect of metformin on bevacizumab-induced changes in plasma levels of vascular endothelial injury markers and proinflammatory cytokines

Then, we sought to investigate whether GDF15 mediates the protective role of metformin against bevacizumab-induced vascular injury and systemic inflammation. H&E staining showed that metformin alleviated vascular endothelial damages in the presence of bevacizumab, whereas siGDF15 administration aggravated the damages compared with siNC (Figure 1B, a-c). In the presence of bevacizumab, siGDF15 injection blocked the protective effect of metformin on endothelial damage (Figure 1B, c-d). Consistently, flow cytometry showed that siGDF15 reversed the inhibitory effect of metformin on vascular endothelial cell apoptosis in the presence of bevacizumab (Figure 1D). Furthermore, siGDF15 treatment overturned metformin-induced suppression in the production of vascular injury markers and proinflammatory cytokines in the presence of bevacizumab (Figure 4A-F). These results suggest that GDF15 mediates the vascular protective and anti-inflammatory properties of metformin.

***siGDF15 suppresses PI3K/AKT/FOXO/PPARγ signaling in the abdominal aorta of mice treated with bevacizumab***

**Figure 5:**
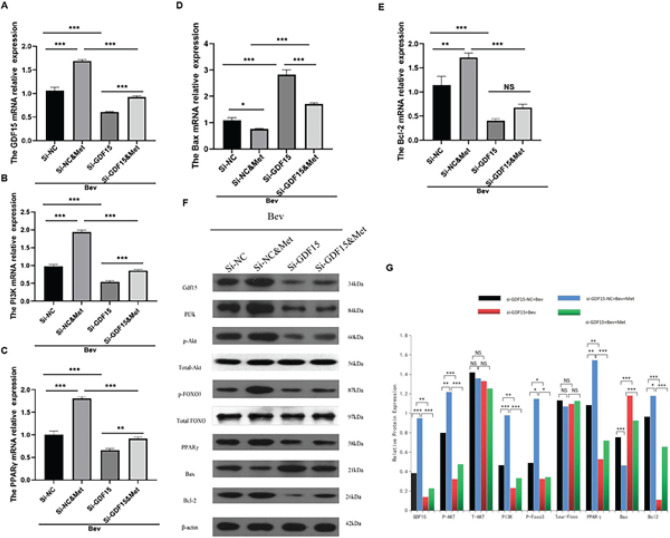
Knockdown of GDF15 reversed the effect of metformin on bevacizumab-induced alterations in the expression of GDF15 and PI3K/AKT/FOXO/PPARγ in mouse abdominal aorta

In order to identify the downstream signaling of GDF15 *in vivo*, we determined the expression of PI3K/AKT/FOXO/PPARγ signaling and apoptotic markers in mouse abdominal aortic tissue samples. We found that GDF15 silencing abolished the metformin-induced transcriptional up-regulation of GDF15, PI3K, Bcl-2, and PPARγ and the transcriptional down-regulation of Bax in mouse abdominal aorta exposed to bevacizumab (Figure 5A-E). GDF15 silencing also effectively reversed the promoting effect of metformin on GDF15, PI3K, P-AKT, P-FOXO3, PPARγ, and Bcl-2 protein expression, and the inhibitory effect of metformin on Bax protein expression (all* P<*0.05) (Figure 5F-G). These data suggest that metformin mitigates the impact of bevacizumab on vascular injury and endothelial cell apoptosis through GDF15/PI3K/AKT/FOXO/PPARγ signaling. 

**Table 1:** Primers used in the experiment

**Gene**	**Forward primer (5'-3')**	**Reverse primer (5'-3')**
GDF15	GGAGCGACTGGGTGTTGT	AGGCGTGCTTTGATCTGC
PI3K	TGACTTTAGAATGCCTCCG	TCTTGGGTGACACTTACGA
BCL-2	GCTACGAGTGGGATGCT	AGGCTGGAAGGAGAAGA
Bax	GATGCGTCCACCAAGAA	AGTAGAAGAGGGCAACCAC
PPARγ	GTTGATTTCTCCAGCATTTC	TTGATCGCACTTTGGTATT
β-actin	CGTTGACATCCGTAAAGAC	TAGGAGCCAGGGCAGTA

**Table 2.** Antibodies used in the experiment

**Antibody**	**Company/Country**	**Catalog number**	**Dilution **
**Primary antibody**			
Rabbit polyclonal to growth differentiation factor 15 (GDF15)	Abcam (UK)	ab211364	1:3,000
Rabbit monoclonal to phosphoinositide 3-kinase (PI3K)	Abcam	ab32089	1:1,000
Rabbit polyclonal to phosphorylated (p)-protein kinase B (AKT)	Cell Signaling Technology (CST, USA)	9271	1:1,000
Rabbit polyclonal to p-forkhead box O3 (FOXO3)	CST	9466	1:1,000
Rabbit polyclonal to peroxisome proliferator-activated receptor γ (PPARγ)	Proteintech (USA)	16643-1-AP	1:800
Rabbit polyclonal to B cell lymphoma-2 (Bcl-2) associated X protein (Bax)	Proteintech	50599-2-Ig	1:1,000
Rabbit polyclonal to Bcl-2	Proteintech	12789-1-AP	1:1,000
Mouse monoclonal to β-actin	ZSbio (China)	TA-09	1:2,000
**Secondary antibody**			
Goat Anti-Rabbit immunoglobulin G (IgG) heavy-chain&light-chain (H&L) horseradish peroxidase (HRP)	ZSbio (China)	ZB-2301	1:10,000
Goat Anti-Mouse IgG H&L (HRP)	ZSbio	ZB-2305	1:10,000

(A, C) Mice were untreated (a) or treated with bevacizumab (b), metformin (c), or bevacizumab+metformin (d). (B, D) Mice were administered si-NC+bevacizumab (a), si-NC+metformin+bevacizumab (b), si-GDF15+bevacizumab (c), or si-GDF15+metformin+bevacizumab (d). The mice were sacrificed after 7 weeks of treatment. Hematoxylin and eosin (H&E) staining was performed to examine vascular endothelial injury (A, B). Flow cytometry was used to determine endothelial cell apoptosis (C, D)

GDF15: growth differentiation factor 15; Bev: bevacizumab; Met: metformin; si-GDF15: adenoviral vectors expressing siRNAs against GDF15; si-NC: adenoviral vectors expressing negative control siRNAs

ELISA was conducted to measure plasma levels of CD62E (A), ET-1 (B), TM (C), vWF (D), IL-6 (E), and TNF-α (F). Data are expressed as the mean±standard deviation (SD). *P<0.05; **P<0.01; ***P<0.001

NS: not significant. CD62E: selectin E; ET-1: endothelin-1; TM: thrombomodulin; vWF: von Willebrand factor; IL-6: interleukin-6; TNF-α: tumour necrosis factor-alpha; Bev: bevacizumab; Met: metformin

Quantitative reverse transcription PCR (qRT-PCR) was performed to determine the mRNA expression of GDF15 (A), PI3K (B), PPARγ (C), Bax (D), and Bcl-2 (E). Western blot analysis was conducted to determine the protein expression of GDF15, PI3K, P-AKT, total AKT, P-FOXO3, total FOXO, PPARγ, Bax, and Bcl-2 (F). (G) Quantification of (F). Data are expressed as the mean±SD. *P<0.05; **P<0.01; ***P<0.001

NS: not significant. GDF15: growth differentiation factor 15; PI3K: phosphatidylinositol 3-kinase; AKT: threonine kinase; FOXO: forkhead box, subgroup O; P-FOXO3: phosphorylated forkhead box, subgroup O3; PPARγ: peroxisome proliferator-activated receptor gamma; Bax: BCL2 associated X; Bcl-2: B cell lymphoma 2; P-AKT: phosphorylated-threonine kinase; Bev: bevacizumab; Met: metformin

ELISA was conducted to measure plasma levels of CD62E (A), ET-1 (B), TM (C), vWF (D), IL-6 (E), and TNF-α (F). Data are expressed as the mean±standard deviation (SD). *P<0.05; **P<0.01; ***P<0.001

NS: not significant. CD62E: selectin E; ET-1: endothelin-1; TM: thrombomodulin; vWF: von Willebrand factor; IL-6: interleukin-6; TNF-α: tumor necrosis factor-alpha; Bev: bevacizumab; Met: metformin; si-GDF15: adenoviral vectors expressing siRNAs against GDF15; si-NC: adenoviral vectors expressing negative control siRNAs

Quantitative reverse transcription PCR (qRT-PCR) was performed to determine the mRNA expression of GDF15 (A), PI3K (B), PPARγ (C), Bax (D), and Bcl-2 (E). Western blot analysis was conducted to determine the protein expression of GDF15, PI3K, P-AKT, total AKT, P-FOXO3, total FOXO, PPARγ, Bax, and Bcl-2 (F). (G) Quantification of (F). Data are expressed as the mean±SD. *P<0.05; **P<0.01; ***P<0.001

NS: not significant. GDF15: growth differentiation factor 15; PI3K: phosphatidylinositol 3-kinase; AKT: threonine kinase; FOXO: forkhead box, subgroup O; P-FOXO3: phosphorylated forkhead box, subgroup O3; PPARγ: peroxisome proliferator-activated receptor gamma; Bax: BCL2 associated X; Bcl-2: B cell lymphoma 2; P-AKT: phosphorylated-threonine kinase; Bev: bevacizumab; Met: metformin; si-GDF15: adenoviral vectors expressing siRNAs against GDF15; si-NC: adenoviral vectors expressing negative control siRNAs

## Discussion

This study aimed to explore the vascular protective activity of metformin in the presence of bevacizumab *in vivo*. We demonstrated that metformin alleviated bevacizumab-induced abdominal aortic injury, endothelial cell apoptosis, and systemic inflammation in mice, along with significant up-regulation of GDF15 expression and activation of PI3K/AKT/FOXO/PPARγ signaling in aortic tissue. These effects were diminished by siGDF15 administration, suggesting that GDF15 mediates the vascular protective activity of metformin against bevacizumab *in vivo*. Metformin may reduce the risk of cardiovascular diseases in cancer patients undergoing bevacizumab therapy.

Clinical trials have shown that patients with colorectal cancer undergoing bevacizumab therapy plus chemotherapy have a substantially increased incidence of cardiovascular adverse events compared with those receiving chemotherapy alone (16, 37), suggesting that bevacizumab induces vascular injury. It has been reported that the established clinical dose of bevacizumab increases the apoptosis of human microvascular endothelial cells compared with lower doses (38). Bota *et al*. have shown that bevacizumab treatment induces apoptosis of HUVECs (39). In addition, bevacizumab induces inflammation in breast cancer cell lines and tumor-bearing mice (40). Consistently, our results showed that bevacizumab treatment induced abdominal aortic injury, endothelial cell apoptosis, and systemic inflammation in mice, contributing to the pathogenesis of bevacizumab-induced cardiovascular adverse events in cancer treatment. 

Studies have shown that metformin protects vascular endothelial cells from inflammation, oxidative stress, and apoptosis, inhibiting increased permeability and differentiation of the cells (41-44). In order to overcome the shortcomings of bevacizumab treatment, we explored whether metformin could protect the blood vessels in the presence of bevacizumab. As expected, metformin effectively abrogated bevacizumab-induced histological abnormalities and endothelial cell apoptosis of the abdominal aorta in mice, accompanied by reductions in bevacizumab-induced overproduction of circulating endothelial cell injury markers and proinflammatory cytokines. In addition, metformin reversed the alterations in the expression of apoptotic markers Bax and Bcl-2 in mouse aortic tissue samples. These findings suggest that metformin alleviates bevacizumab-induced abdominal aortic injury, endothelial cell apoptosis, and systemic inflammation in mice. A study showed that metformin down-regulates hyperglycemia-induced autophagy of HUVECs and alleviates endothelial impairment in diabetic mice (45). Li *et al*. reported that metformin suppresses proinflammatory cytokine production in the vitreous of diabetes patients and human retinal vascular endothelium (46). When combined with bevacizumab therapy, metformin significantly improves the disease status of endometrial cancer, elongates the 1-year progression-free survival of metastatic non-small cell lung cancer, and inhibits the growth of ovarian cancer cells *in vitro* and *in vivo* compared with bevacizumab monotherapy (25, 26, 47). Together with ours, these studies suggest that bevacizumab and metformin, in combination, are superior to bevacizumab monotherapy for many cancers.

GDF15 is highly associated with the vascular system. Overexpression of GDF15 reduces high-fat diet-induced atherosclerotic lesions in apoE-knockout mice (28). In the late stage of atherosclerosis, GDF15 prevents atherosclerosis by inhibiting monocyte chemotaxis and macrophage activation (48). GDF15 reduces damage and apoptosis of endothelial cells caused by high glucose through the PI3K/Akt/eNOS pathway (49). Studies revealed the correlation between metformin and GDF15 expression (31, 50, 51) and the role of PI3K/AKT signaling downstream GDF15 (52, 53). Thus, we investigated the effect of GDF15 silencing on the vascular protective activity of metformin and the alteration in PI3K/AKT/FOXO/PPARγ signaling. We found that siGDF15 abolished the effect of metformin on aortic histological abnormalities, aortic endothelial cell apoptosis, systemic inflammation, and PI3K/AKT/FOXO/PPARγ activation in mice exposed to bevacizumab treatment. Therefore, GDF15 plays an essential role in the protective activity of metformin against bevacizumab-induced vascular injury. To our knowledge, this is the first study to explore the protective role and potential mechanism of metformin in bevacizumab-induced vascular endothelial injury *in vivo*. Nevertheless, the role of PI3K/AKT/FOXO/PPARγ signaling requires further investigation, and the inflammatory status of the vascular endothelium needs to be evaluated.

Bevacizumab is widely used and effective against lung, colorectal, peritoneal, and renal cancer (13, 54), since inhibiting angiogenesis is a recognized strategy against cancer (5). Still, the targets of antiangiogenic drugs (e.g., VEGF) are also involved in the maintenance of normal blood vessels, and such drugs can have detrimental effects on the blood vessels and the cardiovascular risk (9-12), as observed with bevacizumab (14-16, 18, 19). Metformin might possess anticancer effects (22, 23), but its principal interest in cancer management could be the management of endothelial dysfunction (21) induced by cancer treatments like bevacizumab (24). The innovation of this study is the use of metformin to prevent the vascular damage caused by bevacizumab. Therefore, the present study supports the use of metformin in patients treated with bevacizumab to prevent or alleviate the vascular damage caused by bevacizumab. Of course, these effects will have to be examined in formal clinical trials. Metformin might also possess antiangiogenic properties that should be investigated in combination with bevacizumab (55).

## Conclusion

Our study suggests that metformin reduces bevacizumab-induced vascular endothelial injury, apoptosis, and systemic inflammation by activating GDF15/PI3K/AKT/FOXO/PPARγ signaling. Adding metformin is a promising strategy to decrease the risk of cardiovascular diseases in cancer patients treated with bevacizumab. 

## Authors’ Contributions

Li, G, and Ya designed the research study. Li ,Ya, J, F, and La performed the research. Yi and Q provided help and advice on design. Li, C, Ju, and F analyzed the data. Li wrote the manuscript. All authors contributed to editorial changes in the manuscript. All authors read and approved the final manuscript.

## Funding

This research was funded by The Key Science and Technology Research Program of Hebei Provincial Health Commission (No. 20180535, 20211599, 20210065), Key projects of Hebei Science and technology support plan (203777117D), Hebei provincial government-funded specialty capacity building and specialty leader training Project (No. LS201808, LS202101).

## Ethics Declaration

This study and all experimental procedures were approved by the Ethics Committee of the Fourth Hospital of Hebei Medical University (IACUC-4th hosHebmu 2020002) and were carried out in compliance with the ARRIVE guidelines.

## Conflicts of Interest

The authors declare that the research was conducted in the absence of any commercial or financial relationships that could be construed as a potential conflict of interest.
